# *“What counts can’t always be measured”*: a qualitative exploration of general practitioners’ conceptualisation of quality for community pharmacy services

**DOI:** 10.1186/s12875-020-01319-2

**Published:** 2020-11-28

**Authors:** M. Watson, K. Silver, R. Watkins

**Affiliations:** 1 Strathclyde Institute of Pharmacy and Biomedical Sciences, University of Strathclyde, Robertson Wing 601H, 161 Cathedral Street, Glasgow, G40RE Scotland; 2 Department for Health, University of Bath, Bath, England BA2 7AY UK

**Keywords:** General practitioners, Primary health care, Pharmacies, Quality of health care, Qualitative research

## Abstract

**Background:**

The expansion of community pharmacy services is one solution to relieve pressure on general practice in the United Kingdom (UK). There is a paucity of research of general practitioners’ (GPs’) perspectives of quality of care in the community pharmacy sector.

The purpose of this study was to explore GPs’:

- Conceptualisation of quality for community pharmacy services, including the management of acute (low acuity) conditions and defining indispensable aspects of the patient experience (‘always events’)
- Opinions regarding whether and how to measure quality in the community pharmacy sector

**Method:**

Semi-structured interviews were conducted with GPs in the UK. GPs were recruited using the snowballing technique and professional networks. Interviews were audio-recorded, transcribed and analysed using an interpretive approach.

**Results:**

Interviews were completed with 20 GPs from Scotland (*n* = 8) and England (*n* = 12). Multidimensional and inter-related concepts of quality were identified; most dimensions related to patient benefit, as well as impact on GP workload or other health service provision. Interviewees cautioned that *“what counts can’t always be measured”*. GPs’ expectations of quality often mirrored those of their own sector, but were ambivalent about the adoption of a quality outcome framework-type approach. Pharmacist involvement was expected to ensure quality in the management of ‘acute consultations’, however, GPs lacked awareness of community pharmacy personnel type, roles and training. Interviewees’ perceptions of quality varied by pharmacy type; independent pharmacies were sometimes associated with higher quality service delivery than larger chain organisations.

**Conclusions:**

Quality frameworks for community pharmacy services could be partly informed by GP experience and expectations, but need to be contextual to reflect differences between both settings. The importance of person-centred care, consistency and continuity was emphasised together with the need for competent personnel and privacy of interactions.

**Supplementary Information:**

The online version contains supplementary material available at 10.1186/s12875-020-01319-2.

## Background

Each year in the United Kingdom (UK), an estimated 18 million general practitioner (GP) appointments (~ 13% of all consultations) are used for conditions suitable for treatment by community pharmacy personnel, either with non-prescription medicines and/or advice [1]. The suitability of community pharmacies for managing these conditions (hereafter referred to as acute consultations) is gaining recognition and support from primary care organisations and through national policies [2–4]. In England, each community pharmacy serves an average of 4679 citizens [5, 6], the majority (89%) of whom live within a 20-min walk of these services [7]. Many patients prefer to manage acute illness and ailments using self-care [8] with support from community pharmacy personnel.

The Institute of Medicine’s (IOM’s) six dimensions of quality suggest health care should be safe, effective, efficient, equitable, timely and person-centred [9]. An emerging approach to exploring and defining healthcare quality, and one that has also been used in general practice, is the concept of ‘always events’ [10]. The IHI has defined these as “*aspects of the patient experience that are so important to patients, their care partners, and service users that health care providers must aim to perform them consistently for every individual, every time*” [11]. The quality of care from community pharmacies is variable [12, 13] and reassurance is needed that the care provided in this sector is optimised to promote public confidence and GP support. In turn, GP support could enhance confidence in pharmacy service delivery, general awareness, and thus utilisation of services – helping to reduce GP burden [14]. National quality indicators were introduced for community pharmacy in 2017 and have recently been updated in the new 5-year Community Pharmacy Contractual Framework [3], but none refer to the management of acute consultations despite these being regarded as the “*shop window*” of community pharmacy [15].

A research programme was undertaken to explore stakeholders’ perceptions of the quality of community pharmacy services and the management of acute consultations. Precedent studies have been completed with members of the public [16] and pharmacy personnel [15]. The aim of the study reported here was to conceptualise GPs’ perceptions and beliefs about the quality of community pharmacy services in general and, more specifically, using the concept of ‘always events’ and the management of acute consultations. It also considered their attitudes and beliefs about quality measures and indicators for these services.

## Methods

### Study design

Semi-structured interviews with GPs by telephone or in person.

### Recruitment, sampling and consent

The initial participant was recruited through the researcher’s (MW) professional network. Thereafter, snowball recruitment was used [17] with each interviewee being asked to forward an email from the researcher to their GP colleagues/professional networks. On receipt of the email, GPs contacted the researcher if they wished to participate. While necessarily purposive, the intention was to generate a maximum variation sample of up to 30 GPs from different geographical locations (home countries, rural, urban), personal characteristics (age, gender), and practice characteristics (salaried/non-salaried, group/single-handed). Recruitment ceased once theoretical saturation was reached (i.e. when no new themes were identified).

### Data collection

One female researcher (MW), who is a registered pharmacist with over 20 years’ post-doctoral experience of health services research, undertook all data collection. It is possible that the researcher’s pharmacy background may have impacted recruitment, i.e. if GPs were reluctant to engage in pharmacy research. Some participants were known to the researcher; this may have influenced their contribution. Reflexivity on the part of the lead researcher, and the procedure adopted at the analysis stage, aimed to mitigate the impact on findings. The interviews were conducted by telephone or face-to-face between March 2016 and August 2017 and lasted a maximum of one hour. The topic guide (Additional file 1) was informed by existing work on quality and quality improvement, including the concept of ‘always events’, as well as the aforementioned interview study involving pharmacists [11, 15]. During the interview process, the topic guide was refined with (significant) new themes. Participants provided verbal and written consent to participate.

### Data handling and analysis

The interviews were audio-recorded and transcribed verbatim (by experienced transcribers). Field notes were made during the interviews. All transcripts were accuracy checked (by KS/RW) and anonymised prior to being analysed with the support of NVivo 11 software. Interviewees were not asked to review the transcripts.

Thematic analysis was undertaken using themes explored by the topic guide as well with themes derived from the data [18, 19]. Two researchers (KS, RW), from outside the pharmacy profession, with considerable experience of qualitative data analysis, familiarised themselves with the transcripts and coded to broad topic areas (structuring codes). The next (extensive and iterative) phase involved the identification of themes and sub-themes to reflect the research questions (a priori codes/nodes) and from within the data itself (in vivo codes/nodes). Conceptual and cross-cutting themes were identified and coded in addition to relevant topic codes.

Each transcript was coded by one researcher, with most coded by two researchers to ensure reliability. Once coding was complete, a Framework Approach [20] was used to support the systematic analysis around the research questions, to enable an assessment of prevalence and coverage of key themes (i.e. dimensions of quality). Further interpretation and discussion was undertaken amongst the research team to ensure that analytical claims were congruent with the extracts. Participant quotes (anonymised) are presented to illustrate the themes and are designated E or S to indicate England- and Scotland-based interviewees. The study is reported to reflect COREQ criteria [21].

## Results

Interviews were completed with 20 GPs (Scotland *n* = 12, England *n* = 8) and the majority were female (*n* = 12) (Table 1). One interview (E11) was conducted face-to-face and the others were completed by telephone.

**Table 1:** Interviewee Characteristics

ID	Country	Gender (Male (M)/ Female (F))	Number of years working as a GP	Status	Number sessions/week	Practice Size	No. GPs (~WTEs)
1	Scotland	M	20–29	Other	2	11,500	7
2	Scotland	M	20–29	Partner	6	8000	7
3	Scotland	M	20–29	Senior partner	6	11,500	6
4	Scotland	M	10–19	Salaried	6–7	9500	3 (2.25)
5	Scotland	F	30+	Other - OOH	n/a	n/a	n/a
6	Scotland	F	10–19	Partner	6	3500	2
7	Scotland	F	30+	?	8 + 1	< 2200	1.2
8	Scotland	F	0–10	Partner	6	10,000	9 (6.5)
9	England	F	?	Partner (retired)	n/a	n/a	n/a
10	England	F	30+	Partner/Academic		14,700	6 9all p/t + 1 registrar)
11	England	M	0–10	Salaried GP/locum/OOH	6	14,500	13 (not all f/t)
12	England	M	?	Salaried GP/Academic	102	12,000	3 (should have 6) plus locums
13	England	F	10–19	Partner +OOH	7	12,000	7
14	England	M	?	Principal/Academic	2	11,500	6+
15	England	F	?	Partner		30,000	10
16	England	F	20–29	Partner		6500	4 + 2 assistants
17	England	F	30–39	?		8000	10 (some p/t)
18	England	M	20–29	Locum/OOH	n/a	n/a	n/a
19	England	F	20–29	Partner		3050	3
20	England	F	0–9	Locum/Urgent care			

*GP* General Practitioner Other: category added to protect anonymity of interviewee, *OOH* Out-of-hours, *p/t* part-time, *f/t* full-time, *n/a* Not applicable, *?* Missing data

Interviewees’ concepts of quality were inter-related with most dimensions described in relation to patient benefit, as well as others that related to impact on GPs and other health services. Their knowledge of pharmacy services and personnel was frequently gleaned informally and was often incomplete or inaccurate i.e. most interviewees were unsure of the different types of personnel and their roles, and generally lacked familiarity with medicines that were available without a prescription.

### Quality concepts of community pharmacy

When asked what a ‘good’ pharmacy looked like, interviewees offered a broad range of characteristics and distinguished between different types of pharmacies e.g. independent versus large chains. They suggested that pharmacies should be accessible and near to the population that they serve; and should have extended opening hours (S5) for the convenience of patients and known to GPs. Patients should not have to wait “*a long time*” (S6) for services, particularly for prescriptions. Most interviewees said that pharmacies should hold an adequate, well managed stock (S3, S4) of medication (and alternatives) and other medical devices, or be able to obtain them quickly. The provision of a “*timely*”(S8) service was suggested as a way of reducing the need for patients to ‘*yo-yo*’(E10) between the pharmacy and their general practice and would save GP time by avoiding the need to prescribe substitutions. Home deliveries were considered helpful (S8), although there were some concerns around safety i.e. due to lack of direct contact between the patient and pharmacy personnel (E19).

### Person-centred care

Interviewees suggested that a good quality pharmacy would treat patients/customers in much the same way as other health service providers; pharmacists should be visible, available, and offer personalised care, with good listening and communication skills and all staff should have a positive orientation to patients/customers. They should involve people in decisions, treat them with sincerity, and always check their understanding of treatment; they should be kind but not “*paternalistic*” (S8).

*S7: It would have a lot of nice people in it who had enough time to be thorough with their patients, and be not so overworked and stressed that they then can't spend time and be nice to them.*

Some interviewees distinguished between different types of pharmacy and perceived better-quality care to be associated with independent pharmacies, or pharmacies where the pharmacist had greater autonomy.

*E14: A good quality pharmacy would look like an independent quite frankly, and it wouldn’t look like a multiple, and the reason for that is because it responds to patients’ needs, and it responds to the needs with [sic] the local GPs as well…*

### Consistency and continuity of care

Pharmacists should be, and in the experience of several of the GPs were, familiar with patients (particularly elderly) and their conditions and therefore likely to notice health changes, and identify problems relating to drug efficacy, side effects, and compliance, which could then be communicated to the GP. There was an expectation by several GPs that there needed to be continuity of care, helping to build trust with patients (and GPs) but with some acknowledgement that this might be harder to achieve in multiples with large numbers of staff, in urban areas with numerous pharmacies, or in rural areas with few.

*E20: .. if you have got a pharmacist that knows the patient well, knows what usually goes in their Dosette box, is alerted to any changes which might have occurred, either intentionally or by mistake, possibly following discharge and they are aware and they have that insight then it’s really useful, because they know how things run and they know what patients are used to.*

The need for greater standardisation of practise was also recognised to provide reassurance to GPs regarding the quality of care they could expect for their patients.

*E18: I thinks it’s very variable because I ..it can be excellent, particularly where you have situations where you have got regular, high calibre pharmacists who can, who you know the level of service that they are routinely willing to offer. But so often you end up with, particularly with chains and multiples, where you have the reliance on agency or part time staff and therefore the consistency of offering is relatively low. (…) The reason that we don’t [refer to pharmacies] is that we never quite know .. which pharmacies are going to have their A-team on at any given time.*

GPs did not report that they wanted to know *which* specific person was treating their patients, and this was not probed.

### Quality management of acute consultations

Using the concept of ‘always events’, interviewees were asked to describe should *always* and *never* happen during acute consultations (Table 2).

**Table 2:** Always and Never Events and Acute Consultations

Theme	Always	Never
Introduction/name/role	*“introduce yourself, always make it clear who you are, what your role is, so that there is no misunderstanding with the patient as to what the limits of your ..experience are” (S2)*	
Competency – being aware of own competency	*“it’s about being aware of...recognising your limits of competence” (E14)*	*“shouldn’t be done by untrained individuals ...never working outside your competence” (E11)*
Pharmacist involvement	*“it’s not appropriate for someone other than a very experienced pharmacist to actually conduct that consultation ...and it probably needs to be run more like a proper consultation” (S5) “I think it should be done by the pharmacist and not by other staff in the pharmacy” (E19).*	
Information gathering -history taking	*“they [the patient] need to be given a minute..to actually tell their story rather than be asked a lot of direct questions” (S5) “always take a history” (E16) “making sure that and accurate history is taken, especially your allergy history” (S4) “getting a history of co-morbid conditions and concomitant medications” (S1) “self-prescribed medication and GP-prescribed medication” (E9) “the pharmacist should always have access to notes,,,it’s impossible to grasp a full history from a few minutes with the patient” (E11)*	*“jumping straight into prescribing a medication without understanding the context, particularly in terms of current medication, pre-existing ill health” (E12) “just selling ...without a history and without and explanation” (E19) “we never have any consultation without first of all establishing who the patient is and getting their background” (E18)*
Diagnosis	*“always listening to the patient and exclusion of..anything more significant with any particular symptoms” ..(E17)*	*“never be dismissive of patient’s concerns” (E20)*
Safety – Red flags	*“the pharmacist needs to have the ability and the training and the knowledge to ask specific questions and exclude any red flags” (S5) “exclude red flags relating to the condition” (E20)*	*“ideally not miss a red flag”*
Safety – safeguarding	*“anything from an adult protection, vulnerable adults or child protection, ...follow GMC guidance on ..examining patients” (S8)*	
Privacy	*“always have privacy” (S5)*	*“things that pertain to people’s health ....should never be done in a public place” (S5)*
Confidentiality	*“the confidential nature of the consultation is really important” (S1) “you’ve got to have confidentiality” (S 3) “there should always be confidentiality” (S8)*	*“share information inappropriately” (S3)*
Information/advice provision	*“as simple a treatment plan as possible, keep it as simple and straightforward as possible ..ideally I would want some form of teach back or checking the patients has understood” (S 1) “get the right medication; always need to be explained to the patients exactly what to do” (S 5)*	*“patients should never be given things without people fully understanding they fully know how to take them” (S 5) “they [the patient] should never go away from the pharmacist ..pharmacy..without knowing where to go if things get worse” (S 5)*
Commercialism		*“they’re sold a specific product that is significantly more expensive than perhaps another product that would do the same thing” (S4) “unnecessary prescribing expensive medication that has no benefit (E12)*

Most interviewees deemed a “*proper*” acute consultation to be the same, or similar to a GP-managed consultation: structured, transparent and adhering to safety protocols and guidelines, including safeguarding. Some felt that it could be shorter or more simplified, for example using a pro-forma.

*S6: I'm kind of taking it for granted that they've got some kind of framework for safety and protocols or guidance that they work within and they should stick to whatever guidance they're supposed to work within.*

History taking (including current conditions), medications and allergies, was deemed to be essential information. The GPs expected pharmacists to ask the “*right*” diagnostic questions but not a “*barrage*”(E15) of questions, to make an “*appropriate*” diagnosis (possibly with an examination), and offer “*appropriate*” treatment options, whether medication, self-care, or just reassurance.

*E18: ..most* [consultations] *are benign*... *there will be people who will turn up to the pharmacies as the first point of call … this is the one opportunity to get somebody really quite important right, and to redirect them if they are very sick, or to take a step back and manage things very carefully......... it’s not appropriate for somebody other than a very experienced pharmacist to actually conduct that consultation, and it probably needs to be run more like a proper consultation, knowing the patient and …taking and recording a more detailed history, because of the complexity of the patient.*

Pharmacist competence was described in relation to training and qualifications, experience, broad knowledge (not limited to medication) and competency in diagnosis. Most interviewees assumed that acute consultations were managed by pharmacists and considered them to be the most appropriate member of staff to do so. They were generally unaware of the training that staff, including pharmacists, received. Some interviewees expected a high level of skill in diagnosis, including the ability to conduct physical examinations. Others were emphatic that pharmacists are “*not diagnosticians*” (S6) and should not be expected to be; that they would not necessarily have relevant skills, training or experience to diagnose effectively, especially “*undifferentiated*” illness, serious or complex cases. As a minimum, pharmacists were expected to be aware of “*red flags*” (E20) and work within their competencies.

*S2: ..a red eye 99 times out of 100 will be conjunctivitis but it might be acute glaucoma and if you miss that somebody loses the sight in their eye and that's going to be a problem. So I think you need your most senior people dealing with these difficult things.*

*E11: My experience with pharmacists within community … is they’ve not got the skill base to do safe assessments..*

*E15: .. the pharmacist needs to be trained in patient-centred skills, .. I think a lot of the people coming in will know what they want, ask for a product and expect it to be a transaction as you would if you were buying groceries from a shop, and not expect that barrage of questions. (...) .. the danger is if community pharmacists ask too many questions you just end up lying, just to get something*

There was an expectation that pharmacists would refer patients to a GP or other health care provider if necessary, and “*safety-net*” by letting the patient know what to do in the case of no improvement or exacerbation of the condition. However, there were varying opinions regarding what constitutes an appropriate referral, and GPs’ experiences were mixed. Some felt that pharmacists referred to them too often and others were concerned that they might not do so enough.

*S6: .. they shouldn’t just be giving people advice just to get them away out of their doors, or giving them medicines without having appropriately assessed them. So if they're pressured for time and they don’t have the time to deal with the people then either telling them to come back or not seek other advice that's not necessarily right, but again if they're pressured for time it shouldn’t automatically be oh you'll just have to go to the doctor's surgery but giving people options of what they can do.*

*S7: ..they should always have a clear understanding of their illness and how to deal with it, and how to manage it in the appropriate fashion. And they should always leave feeling positive and reassured about how to do that, rather than feeling more anxious and uncertain about how to deal with their own illness.(…)*

Several GPs talked about the need for pharmacy services to be transparent and accountable, through audit or other mechanisms. There was discussion of the (presumed) existence of, or need for, formal oversight and accreditation.

*E18: So it [a quality consultation] needs to be structured and transparent enough so you can audit those outcomes and see that they are broadly comparable, and the patients are ending up with similar outcomes to those that they would if they saw a non-medical prescriber or a GP (…)*

*E17: ..it would be good if they had a central base, like Royal College of Pharmacists or something just to download – ‘this is a specimen policy’ - so they’re all kind of working to the same thing. I’m sure the Boots, and the Lloyds and the Superdrugs* [sic] *all have centralised things but – and making sure that their locum pharmacists are all up to date and qualified.*

### Privacy, confidentiality and governance

Privacy for acute consultations was considered essential. Most GPs expected a ‘good*’* pharmacy to have at least one dedicated room, or at least a separate private area, and to take a “*proactive approach”* to offering it to patients/customers. There was a strong feeling that patients/customers should not have to reveal sensitive information where it could be overheard, and that patient confidentiality should be maintained at all times. Several interviewees had witnessed consultations taking place over-the -counter and felt this was inappropriate.

*S5: .. things that are confidential and things that pertain to people's health, other than just giving people simple things for simple problems should always - should never be done in a public place, where a lot of other people should hear it. So it never should be done without privacy.*

While interviewees reported being willing to discuss individual patients and their treatment with pharmacists, and for many this was a mark of good communication and a positive working relationship, there were mixed views around the recording and sharing patient information. Some thought it essential for pharmacists to keep records of consultations, and several assumed that they already did so.

*S1: When I go in, I'm in some way registered with the pharmacy so they have some form of identification system. They have access to my previous information around over the counter or prescribed medications. (…) a person who has had an allergy to a particular substance, they're not getting it again, if they have access to information such as the repeat prescribing, or previous prescribing, that they check that every time.*

Whilst some interviewees expected pharmacies to have access to written medical history, access to patient records was generally considered to be problematic, both ethically and practically, especially for GPs who felt that they had primary responsibility for “*their*” patients.

*E14: (cont.)…and it responds to the needs with the local GPs as well, who are responsible for the patients’ health, because that’s where their medical record resides.*

Some interviewees cited specific concerns around the risk of community pharmacists sharing patient information with commercial organisations.

### Measuring quality

The majority of interviewees were in general agreement that quality should be measured to provide *“the public some assurance”* (S1). However, defining quality and *“what counts”*(E15) was challenging.

*E15: .. .. what counts can’t always be measured. So I think it’s that whole thing of a lady might pick up her prescriptions every week and the pharmacist she knows, and smiles and asks her how she is - well that’s very hard to measure but that may be a regular conversation she’s having each week.*

It was suggested that pharmacies should have a benchmark against which to measure themselves, and an opportunity to learn from good practice elsewhere.

*E20: .. if you all have certain objectives that you need to achieve .. it might make you realise what other practises and other pharmacists do. ..one thing that has come out of looking at GPs being regulated … we weren’t necessarily aware of good practice in other practices, effective ways of doing things, and we have learnt a lot through communication and through working out what’s considered best practice, how we can then improve.*

Many interviewees anticipated disadvantages with quality measurement. These included increased and/or divisive competition, perverse incentives and tension between financial targets and patient care. Several GPs referred to the “*tick box*”(E17) approach of the Qualities and Outcomes Framework for general practices (QOF) [22] and Care Quality Commission (CQC) [23] inspections, which they suggested did not adequately address overall quality of care.

*E11: I don’t know what the targets would be necessarily and typically if you start entering targets and it becomes a financial incentive and then when you hear about [large pharmacy chain] doing NMRs [sic MURs, medication use reviews] on each other ... I get a bit more sceptical about the use, whether it’s actually for patient focus and quality or is this just another measure of working out how much money do people get.*

### Methods of quality measurement

Interviewees suggested a range of methods for measuring quality (Table 3). Many made comparisons with commercial organisations and tools including Trip Advisor ratings, Amazon customer feedback and food hygiene ratings.

**Table 3:** Methods for quality assessment suggested by interviewees

Customer feedback	Instant feedback devices in pharmacy In-consultation feedback Customer satisfaction survey Friends & family test [28]
External evaluation	Metrics and data audit, including outcomes and safety Comparison with other pharmacies / health services
Peer assessment	Peer support and informal feedback /review 360° appraisal or “Multi System Feedback” (including by GPs and other related professions)
Training and self-assessment	Professional development activities Competency framework for all pharmacists

A few GPs were moderately positive about accreditation and ratings if done in the “*right*” way.

*S1: I'm a big fan of accreditation systems for two reasons, one the evidence is that it drives up quality if done in a particular way… And secondly, it obviously gives the public some assurance that the professional they're dealing with has complied with a set of standards. It gives them some, as I say, assurance rather than just some reassurance or comfort.*

Interviewees were often generally critical of this type of approach and suggested that pharmacies were different from other commercial enterprises, and that services were variable due to different health needs.

*S4: ...if you're talking about a customer-based Trip Advisor kind of rating then...I would be absolutely against that much as I am for general practice because it's just so open to abuse.*

Internet-based ratings were thought less accessible to some patients, unrepresentative and open to manipulation (gaming). There were also concerns about unintended consequences including increased risk aversion in pharmacies, more siloed working, or pharmacies becoming too popular – leading to higher referrals to GPs.

*S8: ..the idea of having a pharmacy .... accredited feels hugely dangerous because .....that becomes very open and becomes a marketing device. Whereas if it's the practitioners [i.e. professional assessment], there is then the accountability of the individual practices in the face of their professional body to maintain those skills and competencies.*

There was discussion of a service level approach to accreditation, with ‘core’ services to ensure continuity, and highlighting of additional services.

*S2: I think it’s better that we raise the consistent standard rather than you know put pharmacy against pharmacy.*

It was suggested that pharmacists are best-placed to decide how the quality of their services should be measured.

*E14: ..start with the patient centeredness argument ..get professionals to reflect upon their own performance based upon how they’re perceived by their professionals, and the people they serve - the patients. So that’s the best starting point. .. ...the only metric or measurement is around professionalism, which is autonomy, accountability and responsibility and that’s what I was talking about with things like appraisal, 360 feedback etc etc rather than bean counting of how many patients have had their blood pressure measured.*

## Discussion

### Summary

To our knowledge, this is the first exploration of GPs’ perceptions and beliefs specifically regarding the quality of community pharmacy services in general and the management of acute consultations in particular. Quality tended to be considered through the ‘lens’ of general practice. Multiple dimensions of quality were identified. The importance of person-centred care, consistency and continuity was emphasised. The interviewees highlighted the importance of acute consultations being managed by competent individuals and with the assurance of privacy to ensure confidentiality and good governance. There was general support for the need to measure quality and the appropriateness of different methods of measurement.

### Strengths and limitations

A diverse range of GPs participated in this study and as such, our results are likely to include and reflect a wide range of GP opinions and concepts on the topics of interest. However, as with all qualitative research, our participant sample was not intended, and cannot be taken to be, representative of all GPs. The interviewees engaged well with the ‘always events’ concept. Due to the recruitment strategies used, a response rate could not be calculated as the denominator was unknown. Data collection was undertaken by one experienced health services researcher (MW) who did not actively disclose her pharmacist background unless specifically asked (although correspondence with GPs included her professional qualifications). The interviewer was known to several interviewees, either directly (S1, E12) or as a result of academic affiliations (E14). The wide range of topics covered indicates that the interviewees shared positive and negative opinions of community pharmacy despite the interviewer’s background. We did not specifically assess whether data saturation was achieved, however, we are confident that *theoretical* saturation was achieved as all recurring themes were identified and no new major themes emerged during the later interviews.

### Comparison with existing literature

To our knowledge, this is the first exploration of GPs’ perceptions and beliefs specifically regarding the quality of community pharmacy services in general and the management of acute consultations in particular.

Quality tended to be considered through the ‘lens’ of general practice as well as each GP’s personal experience of using community pharmacies, rather than professional interactions. The GPs’ perceptions of quality were often aligned to those of the public [16], particularly in terms of relational aspects of consultations and the importance of privacy.

Contrary to the GPs’ perception, only around one third of acute consultations involve a pharmacist [24, 25] and record keeping does not occur routinely. The lack of documentation for pharmacy services has been cited as a barrier to the referral of patients by GPs to community pharmacy services [26]. Low rates of pharmacist involvement could reduce the likelihood of GPs referring patients for pharmacy management. Earlier research showed higher rates of guideline compliant outcomes with acute consultations when pharmacists were involved (driven by greater information gathering) compared with non-pharmacist personnel [27]. Whilst GPs in the current study expected a complete history to be gathered during acute consultations, they cautioned against a “*barrage*” of questions. Pharmacists have identified the ongoing challenge of providing person-centred care with incomplete information as well as respective patient autonomy [15].

A recent systematic review concluded that quality-driven incentives are needed to improve community pharmacy services in general [28]. Whilst it has been suggested that service specifications would enhance service quality [27], none exist for the management of acute consultations in this sector. The GPs in this current study were varied in their support for the introduction of quality measures for community pharmacy services.

### Implications for research and/or practice

Greater collaborative working between GPs and local community pharmacists could achieve better understanding of pharmacy services in general and more realistic expectations in terms of their delivery, however, incentives are needed to achieve this goal [28]. The co-production of service specifications by stakeholders of community pharmacy services could be undertaken as a quality improvement initiative in this sector.

## Conclusions

Quality frameworks for community pharmacy services could be partly informed by GP experience and expectations, but need to be contextual to reflect differences between both settings. The importance of person-centred care, consistency and continuity was emphasised together with the need for competent personnel and privacy of interactions.

## Supplementary Information

**Additional file 1.** Topic Guide.

**Additional file 2.** Additional Illustrative Quotes.

## Data Availability

The data collected for this study have not been made available for sharing because consent for sharing was not requested from participants and resources are not available to de-identify the dataset.

## Abbreviations

COREQ: Consolidated criteria for reporting qualitative research
CQC: Care Quality Commission
f/t: Full time
GMC: General Medical Council
GP: General Practitioner
Id: Identification
IOM: Institute of Medicine
n/a: Not applicable
OOH: Out of hours
p/t: Part time
QOF: Quality Outcomes Framework
UK: United Kingdom
