# Supplementary material for: “What counts can’t always be measured”: a qualitative exploration of general practitioners’ conceptualisation of quality for community pharmacy services
Source: BMC Fam Pract. 2020 Nov 28;21:244. doi: 10.1186/s12875-020-01319-2 (PMC7700702; doi:10.1186/s12875-020-01319-2)
Supplement: Supplementary file 1 — Additional file 1. Topic Guide. [file 12875_2020_1319_MOESM1_ESM.docx]

**Appendix 1: Topic Guide**

This study is exploring GPs’ perceptions and attitudes towards community pharmacies in general and the management of what “acute consultations” in particular. By acute consultations, I mean when someone visits a pharmacy to either buy a medicine that they have requested by name, or when they seek advice for the treatment of a condition or symptom.

**Quality and Community Pharmacy**

Describe your general perceptions of and attitudes towards the role of community pharmacies

- in general
- management of acute consultations specifically

How would you define a “quality pharmacy”?

**Quality and Self Care Consultations**

Let’s move on to discussing acute consultations.

What would a good quality acute consultation look like?

What do you think should *always* happen in self care consultations?

What should *never* happen in self care consultations?

**Quality System for Community Pharmacies**

Some countries use rating systems for their healthcare providers e.g. in England, there is a rating system for general practices and in the US, a rating system is going to be introduced for community pharmacies.

- What are your thoughts about having an accreditation system to reflect the quality of service delivered by community pharmacies?
- How would you use an accreditation system e.g. star rating system?
- If you saw that a pharmacy had a 2-star rating, how would you feel? How about if you saw that a pharmacy had a 5-star rating, how would you feel?

Other things to think about?

Questions for me?
