# Supplementary material for: “What counts can’t always be measured”: a qualitative exploration of general practitioners’ conceptualisation of quality for community pharmacy services
Source: BMC Fam Pract. 2020 Nov 28;21:244. doi: 10.1186/s12875-020-01319-2 (PMC7700702; doi:10.1186/s12875-020-01319-2)
Supplement: Supplementary file 2 — Additional file 2. Additional Illustrative Quotes. [file 12875_2020_1319_MOESM2_ESM.docx]

**Appendix 2: Additional Illustrative Quotes**

| **Theme** | **Quote** |
| --- | --- |
|  |  |
| Knowledge of pharmacy staff | *E17: There’s dispensing assistants, but again it’s often not necessarily those who*  *are seen by the public, or it may be - I don’t know because they don’t necessarily*  *have a badge on giving their role.* |
| Privacy and confidentiality | *E9: Saying you know ‘if you’ve got any queries about your prescription then, then please - we can have a confidential talk, we’ve got a room here waiting. So a sort of proactive approach* |
| Professional Service  Opening Hours | *E18: If we knew there was a network of pharmacies around the county who provided a consistent clinical offering, and we knew the hours they offered it, then we would be much more likely to actually direct patients towards them.* |
| Professional Service  Medicine supply | *S6: Well how long do they have to wait, so if somebody...I know myself when I've gone to chemists sometimes there can be a long queue. ...if you've had to wait a long time it might put some people off and they'd just think oh well I'll just go to see the GP instead.* |
| Professional Service  Medicine stock | *E9: ..we prescribed for example some ear drops and then* *the prescription would come back, or the patient would come back and say they are out of stock – now to us well that is great but what have you got in? Or sometimes I prescribed another ear drop and the patient comes back again these ones are out of stock as well.* |
| Professional Service  Delivery service | *S19: I think their relationship with the patient is really good as well, but that it totally lost - the safety aspects are lost with delivering, they just have a delivery service and sales service. And the trouble is that’s what many patients want.* |
| Accreditation/Continuity | *S6: I suppose if pharmacies had to go through specific training and show that they've*  *got enough throughput of these types of patients on a kind of annual basis then there*  *could be some sort of register of accreditation or whatever. I don’t even know if that*  *exists.* |
| Other  Pharmacists and medicine compliance | *E9: I think giving patients information, so dishing out information leaflets and giving patients - signposting patients to resources - community resources, resources on different medical, social and emotional - you know resources that are out there. I think pharmacists could do a lot more on that. I presume because I have never heard of anybody telling me that they have got that sort of information from pharmacists.* |
| Quality Measures  Accreditations systems | *S3: If the accreditation rating genuinely fits with the - again it's like a hotel isn't it? I mean you* *can get a 7 star one because it's got a fancy swimming pool, but if you don’t like swimming no one really cares. You can get a 5 star rating because it offers a minor illness service but if you don’t want the minor ailment service, you just want to do the basics well, it really doesn't matter.* |
| Quality Measures  Accreditations systems | *S18: I think if they [rating/accreditation schemes] are structured sensitively enough to create an element of competitiveness and to drive practices to offer broader and more consistent services that’s great, but the risk is actually that it does the opposite and that you end up actually making any clinician more conservative in what they are prepared to do because they don’t want to risk being downgraded. So rating surgeons is the best example, the risk is if you rate surgeons..they shy away from doing more risky operations because they don’t want to ruin their stats.* |
| Quality Measures  Accreditations systems | *S2: I wouldn’t be particularly keen on a star system - the same applies to general practice, you want consistent core services to be provided at every location. And there maybe some bolt-on services that are provided in a smaller number of locations. So, you wouldn’t say ‘I'm a 4 star and they're a 2 star’. You would say ‘well I'm a core pharmacy provider but in addition we provide palliative care pharmacy, and we provide Minor Ailment Services with confidential consulting areas’. What they [league tables] focus on which might not always be the most important thing and that then diverts attention away from things that are less easy to measure. So I think it’s better that we raise the consistent standard rather than you know put pharmacy against pharmacy.* |
| Good quality OTC consultation | *E13: [a good quality consultation] Being able to explain their symptoms, being asked particular questions to try and exclude anything more serious, and then advise either to see the doctor if necessary or to have an over the counter medication, or actually not be sold anything just say ‘take some paracetamol and drink plenty of fluids’, but with a safety net if it doesn’t get better in however long, or ‘this happens then go and see your GP’.* |
| Pharmacist Competence  Management of OTC consultations | *E11: My experience with pharmacists within community … is they’ve not got the skill*  *base to do safe assessments and it’s that - you mentioned minor ailments and acute*  *iness. … I’d say minor ailments is non-serious illness and that’s the difficulty with*  *some of the pharmacy training is that they are not taught about all the serious illness.*  *It’s all about what you don’t know, you don’t know what you don’t know - you really*  *aren’t truly doing [a] proper, thorough consultation and that’s mainly what I do in my*  *day job, especially on duty days every patient sits in front of me and I think about*  *what significant illness might I need to exclude with them, and what questions should*  *I ask and what examinations should I do and this could be even minor things like a*  *sore throat and I think pharmacists don’t quite appreciate that. And that’s particularly*  *from their perspective in what they encounter ‘cause they wouldn’t necessarily see*  *all the throat cancers and the things we do, and they wouldn’t necessarily see the*  *really sick people that I have in A&E and how people deteriorate and people*  *collapsing in my surgery and things.* |
| Pharmacist Competence  Management of OTC consultations | *S6: .. its difficult as a GP to think about other people doing aspects of your job that you've done ad nauseum day in day out, and don’t necessarily give much of a thought to it because that's what you've been trained to do. But the thing is, sometimes I think pharmacists are trying to wear too many hats, but they're trained in one area that's fantastic, but they're not diagnosticians, so they shouldn’t be expected to be diagnosticians.(…) I mean I thinks us GPs we would love people to take the burden of trivial illnesses away from us but it's the difficulty...it's much easier for us to say that's something trivial than it is for somebody who doesn't see that day in day out because a lot of its to do with pattern recognition and the sheer volume of people that you've seen with those kinds of conditions. If pharmacists are not seeing it or they're not confident or...they've not built up the experience, then it's difficult for them to necessarily do that. I don’t know how you kind of get around that.* |
| Competence | *E18: I think my current experience is that generally pharmacies are much better at*  *identifying which patients do need to be seen acutely by a GP, and fall outside of*  *their ability to offer self-care advice. But I think there is a concern about consistency*  *at both ends of the spectrum in terms of encouraging people to medicalise conditions*  *that really don’t need any treatment at all by having over the counter medicine, and*  *the threshold at which ‘well I don’t really want to take responsibility for telling this*  *person they’re alright so I am going to push the responsibility onto someone else’.*  *So I think that the thing - I think the more we know there’s consistency the more A)*  *the more likely we are to direct patients towards pharmacies, but also [B] the more*  *comfortable and willing we are about receiving referrals from pharmacies without*  *any triage. I think it’s about creating a strong sense of consistency in terms of what*  *the standards are and what the thresholds for referral are etc. that works in both*  *directions.* |
